# Expression of mutant TDP-43 induces neuronal dysfunction in transgenic mice

**DOI:** 10.1186/1750-1326-6-73

**Published:** 2011-10-26

**Authors:** Ya-Fei Xu, Yong-Jie Zhang, Wen-Lang Lin, Xiangkun Cao, Caroline Stetler, Dennis W Dickson, Jada Lewis, Leonard Petrucelli

**Affiliations:** 1 Department of Neuroscience, Mayo Clinic, (4500 San Pablo Road), Jacksonville, (32224), USA; 2 Center for Translational Research in Neurodegenerative Disease (CTRND), College of Medicine, University of Florida, (1275 Center Drive), Gainesville, (32610), USA; 3 Department of Neuroscience, College of Medicine, University of Florida, (1275 Center Drive), Gainesville, (32610), USA

**Keywords:** aggregation, ALS, mitochondria, mouse model, tau

## Abstract

**Background:**

Abnormal distribution, modification and aggregation of transactivation response DNA-binding protein 43 (TDP-43) are the hallmarks of multiple neurodegenerative diseases, especially frontotemporal lobar degeneration with ubiquitin-positive inclusions (FTLD-U) and amyotrophic lateral sclerosis (ALS). Researchers have identified 44 mutations in the *TARDBP *gene that encode TDP-43 as causative for cases of sporadic and familial ALS http://www.molgen.ua.ac.be/FTDMutations/. Certain mutant forms of TDP-43, such as M337V, are associated with increased low molecular weight (LMW) fragments compared to wild-type (WT) TDP-43 and cause neuronal apoptosis and developmental delay in chick embryos. Such findings support a direct link between altered TDP-43 function and neurodegeneration.

**Results:**

To explore the pathogenic properties of the M337V mutation, we generated and characterized two mouse lines expressing human TDP-43 (hTDP-43_M337V_) carrying this mutation. hTDP-43_M337V _was expressed primarily in the nuclei of neurons in the brain and spinal cord, and intranuclear and cytoplasmic phosphorylated TDP-43 aggregates were frequently detected. The levels of TDP-43 LMW products of ~25 kDa and ~35 kDa species were also increased in the transgenic mice. Moreover, overexpression of hTDP-43_M337V _dramatically down regulated the levels of mouse TDP-43 (mTDP-43) protein and RNA, indicating TDP-43 levels are tightly controlled in mammalian systems. TDP-43_M337V _mice displayed reactive gliosis, widespread ubiquitination, chromatolysis, gait abnormalities, and early lethality. Abnormal cytoplasmic mitochondrial aggregates and abnormal phosphorylated tau were also detected in the mice.

**Conclusion:**

Our novel TDP-43_M337V _mouse model indicates that overexpression of hTDP-43_M337V _alone is toxic *in vivo*. Because overexpression of hTDP-43 in wild-type TDP-43 and TDP-43_M337V _mouse models produces similar phenotypes, the mechanisms causing pathogenesis in the mutant model remain unknown. However, our results suggest that overexpression of the hTDP-43_M337V _can cause neuronal dysfunction due to its effect on a number of cell organelles and proteins, such as mitochondria and TDP-43, that are critical for neuronal activity. The mutant model will serve as a valuable tool in the development of future studies designed to uncover pathways associated with TDP-43 neurotoxicity and the precise roles TDP-43 RNA targets play in neurodegeneration.

## Background

TDP-43 is the major component of ubiquitinated inclusions in most cases of ALS and FTLD-U [1,2], and the link between TDP-43 mutations and neurodegeneration was first established in 2008 [3,4]. Autosomal dominant mutations in *TARDBP*, the gene encoding TDP-43, are associated with sporadic and familial ALS [3-7]. TDP-43 is a ubiquitously expressed 414-amino acid nuclear protein and a highly conserved heterogeneous nuclear ribonucleoprotein (hnRNP). TDP-43 has high-binding affinity for the (TG)_n _motif and is involved in gene transcription, pre-mRNA splicing, mRNA stability, and mRNA transport [8,9]. Under disease conditions, TDP-43 is truncated, phosphorylated, ubiquitinated and aggregated both in the nucleus and cytoplasm. Under such conditions, cytoplasmic TDP-43 aggregation coincides with the depletion of nuclear TDP-43. The manner through which TDP-43 causes neurodegeneration has not been identified; however, recently TDP-43 mouse models generated by our group and others demonstrate that overexpression of TDP-43 [either wild-type, A315T mutant, G348C or ΔNLS (defective nuclear localization signal TDP-43)] is toxic and can cause neurodegeneration in the central nervous system [10-16]. That being said, the various TDP-43 transgenic models exhibit other similarities as well as differences. For instance, most transgenic models showed increased ubiquitin levels, TDP-43 fragmentation, phosphorylation, gliosis, motor functional impairments, and shortened lifespan. On the other hand, neuronal loss, caspase activation, redistribution of TDP-43 from nuclei to cytoplasm, cytoplasmic TDP-43 inclusions, down regulation of endogenous mTDP-43 and abnormal mitochondrial aggregation were either only seen in some of those TDP-43 transgenic mice, or not examined. To further confirm the toxic effect of TDP-43 overexpression and to specifically study mutant TDP-43, we generated transgenic mice expressing hTDP-43_M337V _under control of the mouse prion (PrP) promoter [17].

In the TDP-43_M337V _mice, hTDP-43_M337V _is mainly expressed in the brain and spinal cord, a finding that is consistent with transgenic mice overexpressing wild-type TDP-43 that we previously reported [11]. TDP-43_M337V _mice exhibited certain features similar to those seen in ALS, such as TDP-43 cleavage, phosphorylation, aggregation, increased ubiquitination, gliosis, gait disturbances, and early lethality; however, the mice also exhibited other features not yet reported in humans, which may be due to the effect of hTDP-43_M337V _overexpression. Such features include: down regulation of mTDP-43, abnormal mitochondrial aggregation and abnormal tau phosphorylation.

## Results

### Generation of transgenic mice overexpressing hTDP-43 _M337V _protein

**Figure 1:**
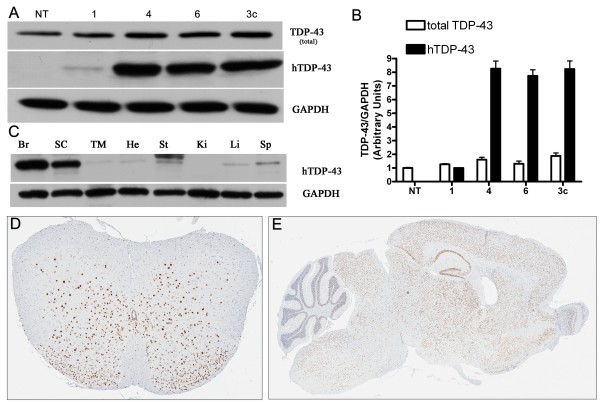
**TDP-43 expression in TDP-43 M337V mice**. (A) Western blots of brain lysates from non-transgenic (NT) mice, founder line 1, 4, 6 of TDP-43_M337V _hemizygous mice and line 3c of TDP-43_WT _hemizygous mice show that TDP-43_M337V _(lines 4, 6) and TDP-43_WT _mice are expression matched for both total (mTDP-43 and hTDP-43) and hTDP-43 levels, which was subsequently confirmed (B) by densitometric measurements. Compared with NT mice (1 ± 0.04), the levels of total TDP-43 in lines 1, 4, 6 and 3c are 1.2 ± 0.05; 1.6 ± 0.07; 1.3 ± 0.17; 1.9 ± 0.22 fold respectively. Compared to line 1(1 ± 0.06), the levels of hTDP-43 of line 4, 6 and 3c are 8.3 ± 1.2; 7.7 ± 1.0 and 8.3 ± 1.3 fold, respectively. Data shown in (B) are means ± SEM of six different mice of each genotype. (C) Western blot of tissue lysates of TDP-43 _M337V _mice from brain (Br), spinal cord (SC), thigh muscle (TM), heart (He), stomach (St), kidney (Ki), live (Li) and spleen (Sp) probed with an antibody against hTDP-43 reveals high expression in the brain and spinal cord (line 4). (D-E) Immunohistochemistry shows hTDP-43 distributed throughout the gray matter of the spinal cord (A) and brain (B) in the hemizygous TDP-43_M337V _mice.

**Figure 2:**
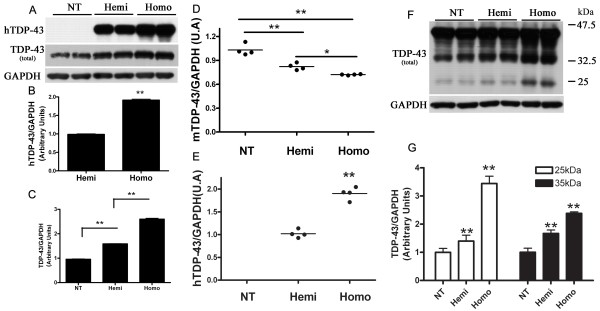
**Down regulation of mouse TDP-43 and production of lower molecular weight (LMW) TDP-43 species in TDP-43_M337V _mice**. Western blots (A) of brain lysates from NT, hemizygous (Hemi) and homozygous (Homo) TDP-43_M337V _mice probed for hTDP-43 or total TDP-43 levels were quantified by (B) densitometry to show that mutant hTDP-43 expression is approximately 90% higher in homozygous mice compared to hemizygous mice (Hemi = 1 ± 0.04; Homo = 1.9 ± 0.06), ** p < 0.001. Densitometric analysis (C) shows that total TDP-43 levels in each genotype group (NT = 1 ± 0.05; Hemi = 1.6 ± 0.03; Homo = 2.7 ± 0.08). ** p < 0.001. Data shown are means ± SEM of six different mice of each genotype. (D) Quantitative real time PCR (qPCR) using mTDP-43-specific primers demonstrate the mRNA levels of mTDP-43 in NT, hemizygous and homozygous mice brain (NT = 1 ± 0.07; Hemi = 0.8 ± 0.05; Homo = 0.7 ± 0.01). * p < 0.05, ** p < 0.01. (E) qPCR using hTDP-43 - specific primers shows the mRNA levels of hTDP-43 in hemizygous and homozygous mice brain (Hemi = 1 ± 0.09; Homo = 1.86 ± 0.14). ** p < 0.01. Data shown in (D) and (E) are means ± SEM of four different mice of each genotype. (F) Darker exposure of the Western blot shown in (A) revealed increased LMW species of TDP-43 in hemi- and homozygous TDP-43_M337V _mice. (G) Densitometric analysis of ~25 kDa and ~35 kDa TDP-43 species indicates that both species correlate with total TDP-43 expression (For ~25 kDa species, NT = 1 ± 0.14; Hemi = 1.4 ± 0.21; Homo = 3.4 ± 0.26. For ~35 kDa species, NT = 1 ± 0.32; Hemi = 1.6 ± 0.28; Homo = 2.4 ± 0.13). ** p < 0.01. Data shown are means ± SEM of six different mice of each genotype.

To explore the pathogenic properties of mutant (M337V) TDP-43, we generated transgenic mice using the mouse prion promoter to constitutively drive expression of full-length hTDP-43 carrying the M337V mutation. Three (Lines 1, 4, and 6) of eight independent founder lines showed germline transmission. The expression level of human TDP-43 was low in line 1 (~20% of endogenous levels), while lines 4 and line 6 had similar hTDP-43 expression levels to that of wild-type TDP-43 mice (TDP-43_WT _line 3c hereafter termed TDP-43_WT_), which we previously generated with the same promoter [11] (Figure 1A, B). Biochemical analyses of hTDP-43_M337V _showed that protein expression was highest in the brain and spinal cord, with low levels in other tissues (Figure 1C). The immunohistochemistry (IHC) of hemizygous mice showed that hTDP-43 _M337V _expression was primarily in nuclei and distributed throughout the gray matter of the spinal cord and brain (Figure 1D, E). Hemizygous mice from all lines were phenotypically, histologically, and immunohistochemically indistinguishable from NT mice up to 12 months of age, currently the oldest age available. Given the similar levels of expression between lines 4 and 6, the full characterization of line 4 is shown and all subsequent description of TDP-43_M337V _mice refer to homozygous mice from line 4 unless otherwise noted. The expression level of hTDP-43_M337V _in the brains of homozygous mice was about 1.9 ± 0.06 fold over that of hemizygous mice (Figure 2A, B). The total levels of TDP-43 protein (human and mouse TDP-43) in the brains of hemizygous and homozygous mice were 1.6 ± 0.03 and 2.7 ± 0.08 fold that of endogenous mouse TDP-43 in the non-transgenic mice (NT), respectively (Figure 2A, C). These results obtained from hTDP-43_M337V _compared with total TDP-43 indicated that endogenous mTDP-43 protein levels were likely down regulated in TDP-43_M337V _mice, in a dose-dependent fashion. We confirmed that mTDP-43 was similarly down regulated at the mRNA level (~ 20% and 30% reduction of mTDP-43, respectively, compared to NT mice) using real time-PCR analyses of hemizygous and homozygous brains (Figure 2D). The mRNA levels of hTDP-43 in the brain of homozygous mice were 1.86 ± 0.14 fold of that in the hemizygous mice (Figure 2E), consistent with the hTDP-43 protein levels. Moreover, the ~25 kDa and ~35 kDa TDP-43 LMW species also increased significantly in the hemizygous and homozygous mice, and their levels correlated with the levels of full-length TDP-43 (Figure 2F, G).

**Figure 3:**
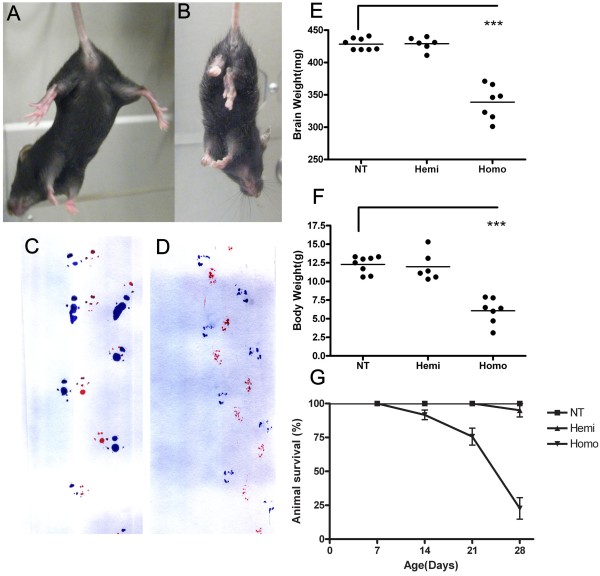
**Reduced brain and body weight and motor dysfunction in TDP-43_M337V _mice**. (A-B) Upon tail elevation, NT mice (A) showed normal escape response by splaying their hind limbs while homozygous TDP-43_M337V _mice (B) held their hind limbs close to their body and failed to show proper escape extension. (C-D) Gait of NT and TDP-43_M337V _mice was evaluated by inked foot placement on paper. 1 month old (C) NT mice show normal foot placement and gait; whereas, (D) homozygous TDP-43_M337V _mice showed an irregular, inwardly-placed foot falls with a dragging pattern. Forepaws and hind paws were coated in red and blue ink, respectively, to evaluate placement of paws during travel. (E) At 1 month, brain weight and (F) body weight of homozygous TDP-43_M337V _mice was significantly lower than that of age-matched NT and hemizygous mice. Brain weight: NT = 428 ± 9 mg; Hemi = 429 ± 11 mg; Homo = 338 ± 29 mg. Body weight: NT = 12.3 ± 1 g; Hemi = 12 ± 2 g; Homo = 6 ± 2 g. Data shown are the means ± SEM of 6-8 mice per group. ***p < 0.0001. (G) Hemizygous TDP-43_M337V _mice were mated, and the survival of the resulting pups of each genotype was determined. The results are plotted as a percentage of pups alive per postnatal day of life. Survival rate for all cohorts were calculated using Kaplan-Meier methods (p < 0.001 for overall log-rank test). Homozygous TDP-43_M337V _mice had higher mortality (about 70% death) around 1 month of age, which was statistically significant compared with NT and hemizygous littermates (p < 0.001).

At approximately post-natal day 21, homozygous TDP-43_M337V _mice began to have body tremors and difficulty recruiting their hindlimbs (not shown). They failed to show proper escape extension by splaying their hindlimbs upon elevation, as shown by their NT counterparts (Figure 3A, B). They displayed an irregular, dragging gait pattern, due at least in part to limb weakness (Figure 3C, D). By ~ 1 month old, homozygous TDP-43_M337V _mice had significantly lower brain and body weight compared with their NT and hemizygous littermates (Figure 3E, F). Due in part to muscle weakness, homozygous TDP-43_M337V _mice were unable to feed from a food hopper, lost the ability to right themselves, became moribund and required euthanasia. Approximately 70% of the homozygous TDP-43_M337V _mice became moribund by 1 month of age, which was statistically significant compared with NT and hemizygous littermates (Figure 3G). We had previous success (unpublished) extending the lifespan of mouse models that showed early lethality through the use of intensive care such as delayed weaning and supplementing diets with gel and dough. We have now employed similar measures in an effort to prolong the lives of homozygous TDP-43_M337V _transgenic mice. The phenotype of the homozygous TDP-43_M337V _mice from line 6 (not shown) was similar to that described for line 4.

### Pathological alteration of TDP-43

**Figure 4:**
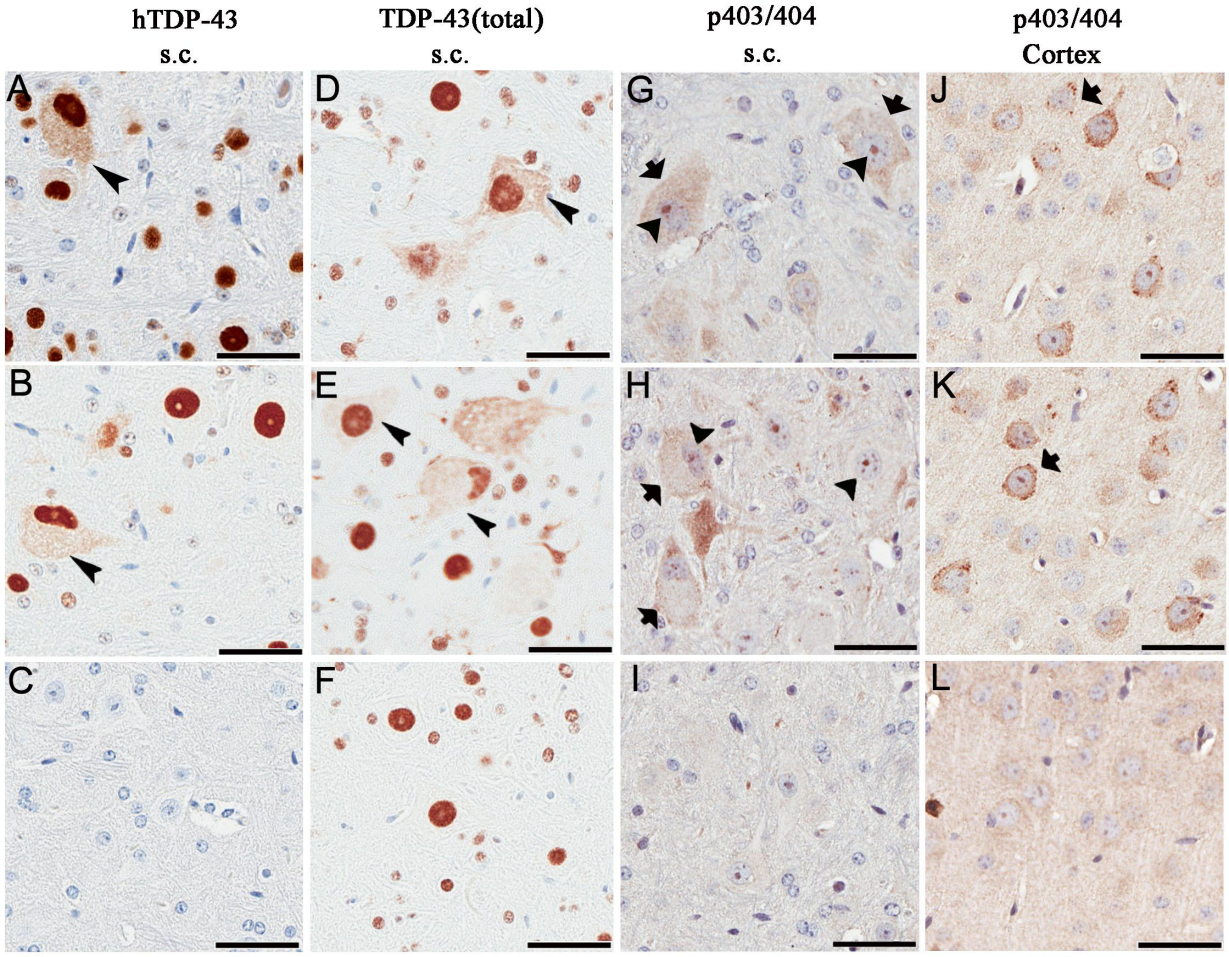
**Distribution of TDP-43 and phosphorylated TDP-43 (pTDP-43) aggregates in TDP-43_M337V _mice**. Immunostaining in spinal cord sections of a 1-month old TDP-43_M337V _from (A) line 4, (B) line 6 and (C) NT mice shows hTDP-43 in nuclei, with occasional cytoplasmic staining (arrowhead); hTDP-43 was not observed in NT mice. (D-F) Immunostaining of spinal cord sections for total TDP-43 shows that TDP-43 immunoreactivity in both nuclei and cytoplasm in TDP-43_M337V _mice (arrowhead) (D-E), but only in nuclei in NT mice (F). Immunostaining of spinal cord (G-I) or cortical (J-L) neurons for phosphorylated (p403/404) TDP-43. (G-H) Nuclear bodies (arrowheads) within spinal motor neurons of TDP-43_M337V _mice immunostained for pTDP-43. Occasionally, diffuse cytoplasmic pTDP-43 was found within the motor neurons in the anterior horn of TDP-43_M337V _mice (arrows). (I) NT mice lacked similar pTDP-43 staining in the spinal cord. (J-K) Cytoplasmic aggregates of pTDP-43 (arrows) were often seen in cortical neurons of TDP-43_M337V _mice that were absent from (L) NT mice. Scale bars: 50 μM.

Immunohistochemistry was performed on both line 4 and line 6 of TDP-43_M337V _and NT mice. The results showed that TDP-43 was mostly in nuclei in the brain and spinal cord of TDP-43_M337V _and NT mice and that the TDP-43_M337V _mice specifically expressed high levels of hTDP-43 that were not seen in the controls (Figure 4A-C). Cytoplasmic TDP-43 was detected with either a human TDP-43 specific antibody or total TDP-43 antibody that recognized both human and mouse TDP-43. Cytoplasmic TDP-43 was detected in spinal cord neurons and less frequently in the brainstem and cortex of TDP-43_M337V _mice (Figure 4A, B, D and 4E arrowhead, Table 1). Abnormally phosphorylated (pS403/pS404) TDP-43 (pTDP-43) was frequently located within nuclear bodies or diffusely within the cytoplasm of motor neurons in the anterior horns of the spinal cord of TDP-43_M337V _mice (Figure 4G, H arrowheads, arrows) but not in the NT mice (Figure 4I). Far less cytoplasmic pTDP-43 was detected in the posterior horn of TDP-43_M337V _mice and in the brainstem (< 5 neurons/section; Table 1). pTDP-43-immunoreactive nuclear bodies were not detected in the brains of TDP-43_M337V _mice (Table 1). Multiple small, distinct, cytoplasmic inclusions that were immunoreactive for pTDP-43 were frequently observed within neurons in layer V of the cortex of TDP-43_M337V _mice (Figure 4J, K, arrows) that were not observed in NT controls (Figure 4L). The extent and distribution of these features in the TDP-43_M337V _mice was similar to that observed in our previously reported TDP-43_WT _mice [11].

**Table 1:** Regional distribution of pathologies in TDP-43_M337V _mice

	Cortex, layer V	Hippocampus	Striatum	Brainstem	Cerebellum	Anterior horn of spinal cord	Posterior horn of spinal cord
Cytoplasmic TDP-43	+	-	-	+	-	++	+

Nuclear pTDP-43	-	-	-	+/-	-	++	+

Cytoplasmic pTDP-43	++	-	-	+	-	++	+

Ubiquitination	++	+	+	++	-	++	++

IBA-1-positive microglia	+	-	-	++	-	++	+

GFAP-positive astrocytes	+	-	-	++	-	++	+

Mitochondrial aggregates	+	-	-	+	-	++	+

p-tau	++	++	+	+	-	+/-	+/-

### Cytoplasmic eosinophilic aggregates, ubiquitination, and gliosis in TDP-43_M337V _mice

**Figure 5:**
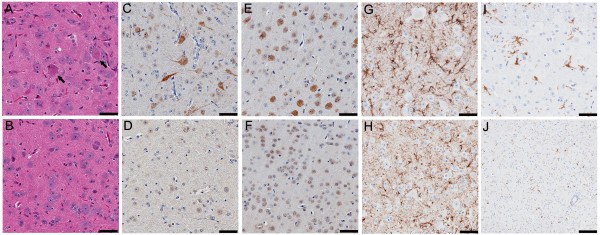
**Neuropathology in TDP-43_M337V _mice**. Hematoxylin and eosin staining revealed (A, arrows) eosinophilic aggregates in spinal motor neurons from TDP-43_M337V _mice that are not observed in (B) NT mice. Abnormal ubiquitin immunoreactivity was present in the cytoplasm and nucleus of neurons in the (C) spinal cord of TDP-43_M337V _mice, but not in (D) NT mice. Similar ubiquitination was observed in the (E) cortex of TDP-43_M337V _mice, but not in (F) NT mice. Enhanced (G-H) GFAP and (I-J) IBA-1 immunoreactivity indicative of reactive astrogliosis and activated microglia, respectively, were observed in TDP-43_M337V _mice (G, I), but not NT mice (H, J). Scale bars: 100 μM.

A striking histological feature in TDP-43_M337V _mice was the presence of cytoplasmic eosinophilic aggregates (Figure 5A) located within the neurons of the anterior horn of the spinal cord and less frequently in the posterior horn and brainstem. These aggregates were absent from NT mice (Figure 5B). Neuronal loss due to apoptosis was not detected in TDP-43_M337V _mice, as assessed by TUNEL staining and staining for activated caspase 3 (data not shown); however, increased ubiquitination and reactive gliosis were observed in TDP-43_M337V _mice and not in NT mice (Figure 5C-J). Ubiquitination was both widespread, and generally increased, in both the nucleus and cytoplasm of neurons in spinal cord and brain (Figure 5C, E). Ubiquitination was more widespread than cytoplasmic TDP-43 or phospho-TDP-43 aggregates (Table 1), and co-immunoprecipitation studies indicated that hTDP-43 was not ubiquitinated (Additional file 1). Reactive gliosis was also seen in the TDP-43_M337V _mice, as GFAP-positive astrocytes and IBA-1-positive microglia were elevated in the spinal cord and brainstem of TDP-43_M337V _mice compared with NT mice (Figure 5G-J; Table 1). Each of these features observed in the TDP-43_M337V _mice was similar to that observed in our previously reported TDP-43_WT _model [11]. Homozygous TDP-43_M337V _mice from line 4 and 6 (not shown) showed similar pathological changes.

### Abnormal mitochondrial aggregates in TDP-43_M337V _mice

**Figure 6:**
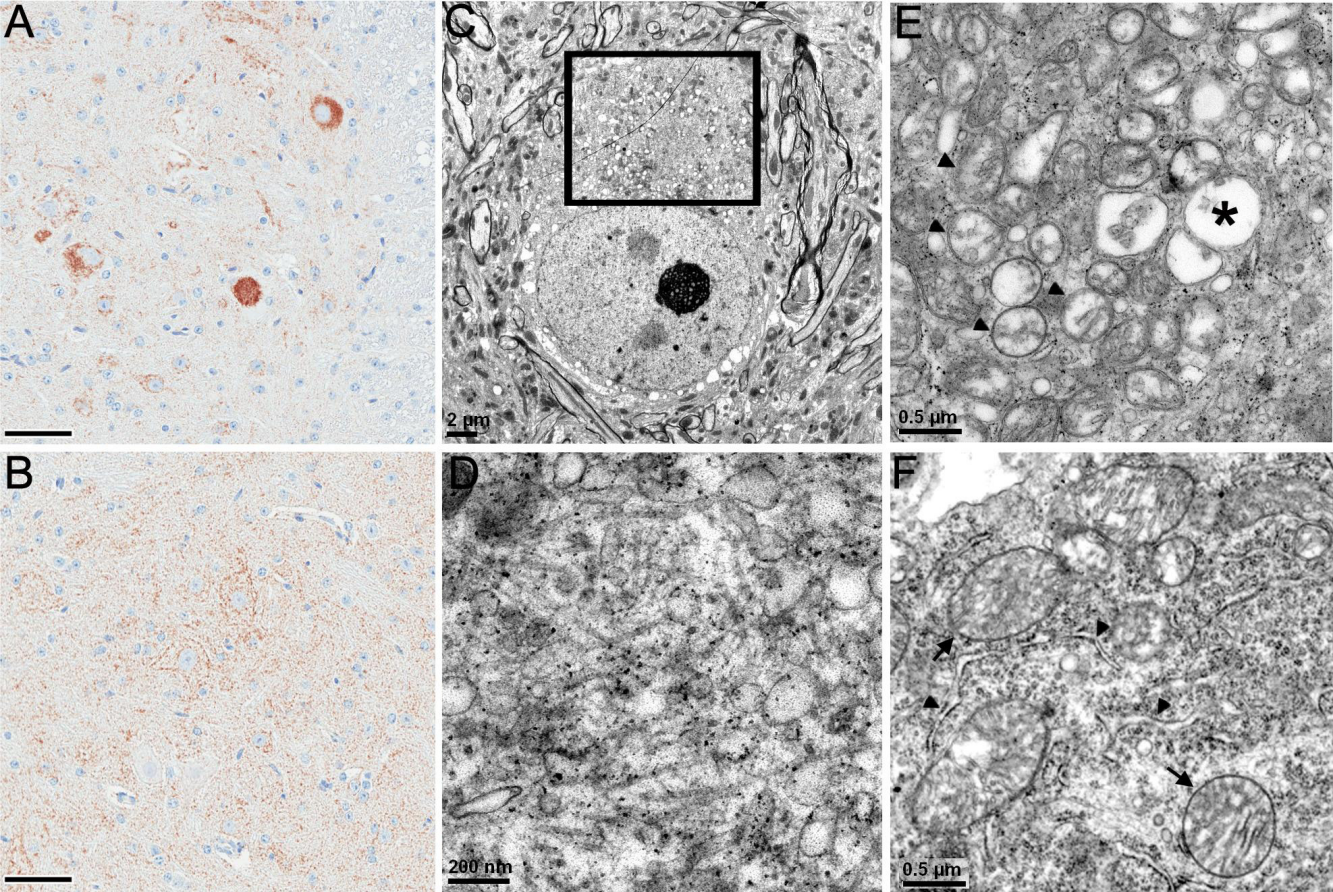
**Abnormal mitochondrial aggregations in TDP-43_M337V _mice**. COX-IV immunoreactivity illustrates densely stained aggregates in a spinal motor neuron of (A) TDP-43_M337V _mice, but not in (B) NT mice. (C) Electron micrograph of a spinal motor neuron from a TDP-43_M337V _mouse shows a cluster of mitochondria surrounding a filamentous core (boxed area). (D) Enlargement of the filamentous core showing 20 nm filaments and various vesicles. Small dense granules are stain precipitates. (E) Electron micrograph of a mitochondrial aggregate in another spinal motor neuron of the TDP-43_M337V _mouse. Note the close proximity of mitochondria with varying degrees of degenerating inner cristae (arrowheads), compared to normal mitochondria in motor neurons of (F) NT mice. (E) The degenerating mitochondria of TDP-43_M337V _mice also appear smaller in size compared with those of (F) NT mice. Asterisk in (E) indicates a vacuolated mitochondrion. (F) In NT mice, mitochondria (arrows) were usually separated by other cytoplasmic organelles, e.g. rough endoplasmic reticulum (arrowheads) and ribosomes. Scale bars: 50 μM in A and B.

The eosinophilic aggregates in spinal motor neurons of TDP-43_M337V _mice were immunoreactive for the mitochondrial marker, COX-IV (Figure 6A), which indicates the aggregates were composed of abnormal clusters of mitochondria. NT mice (Figure 6B) did not show altered redistribution of COX-IV immunostaining into the juxtanuclear pattern observed in the TDP-43_M337V _mice. Electron microscopic (EM) results confirmed that the majority of neuronal cytoplasmic inclusions in the spinal motor neurons of TDP-43_M337V _mice contained aggregates of mitochondria (Figure 6C) that were occasionally surrounded by a core of microtubules (20 nm) in random orientations (Figure 6D). The aggregated mitochondria showed variable degrees of loss of inner cristae and vacuolization (Figure 6E). The degenerating mitochondria in TDP-43_M337V _mice (Figure 6E) were smaller than those in NT mice (Figure 6F). In NT mice, mitochondria were usually admixed with other cytoplasmic organelles such as endoplasmic reticulum and ribosome (Figure 6F); however, this intervening organelles were absent in the neuronal aggregates of TDP-43_M337V _mice (Figure 6E). The mitochondrial aggregates in the TDP-43_M337V _mice were frequent in the spinal cord and brainstem, less frequent in the cortex, and rare in other brain regions (Table 1). Homozygous TDP-43_M337V _mice from line 6 also developed juxtanuclear mitochondrial aggregates (not shown).

### M337V TDP-43 does not alter mitochondrial fusion and fission proteins

We have previously demonstrated that mitochondrial clustering in neurons of TDP-43_WT _mice is accompanied by changes in protein levels and/or phosphorylation of proteins that regulate mitochondrial fission and fusion [11]. Given the similarity between mitochondrial aggregates in TDP-43_M337V _mice and our previously described TDP_WT _mice, we sought to determine if mitochondrial fission and fusion proteins were altered in the TDP-43_M337V _mice. Surprisingly, the alterations to pDLP1 (Ser616), Fis1, and MFN1 that were observed in the TDP-43_WT _mice, regardless of line, were not present in the TDP-43_M337V _mice, despite the comparable mitochondrial aggregation in each transgenic model (Additional file 2).

### Chromatolysis in TDP-43_M337V _mice

**Figure 7:**
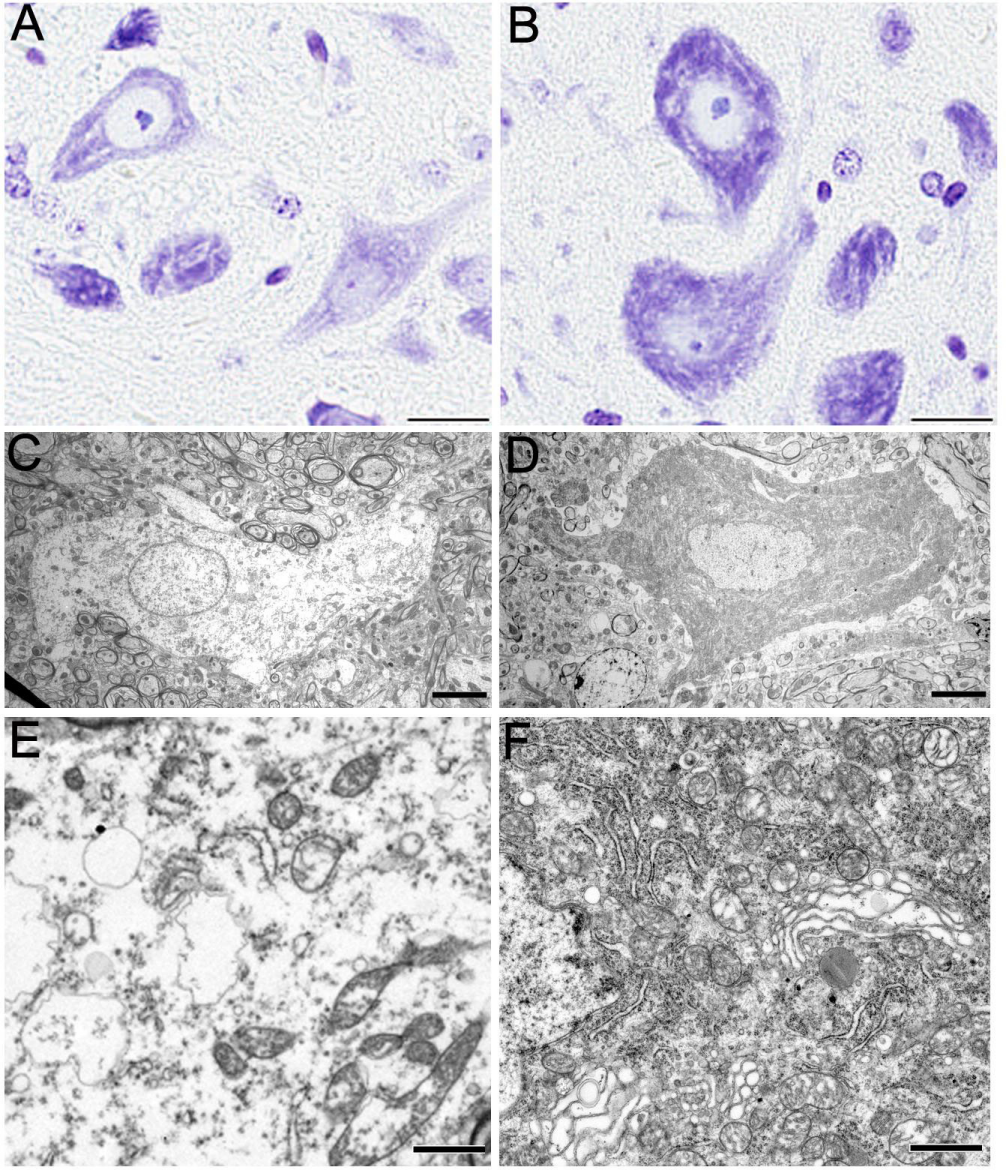
**Chromatolysis in TDP-43_M337V _mice**. Nissl staining of motor neurons of (A) TDP-43_M337V _mice is much weaker than that in (B) NT mice. (C) Electron micrograph of a chromatolytic neuron of TDP-43_M337V _mice compared to the (D) normal neuron of the NT mouse shows rarefaction of cytoplasmic organelles (E), compared with the normal cytoplasmic density (D) and packed organelles (F) of NT mice. Scale bars: 20 μM in A and B; 5 μM in C and D; and 1 μM in E and F.

In the spinal motor neuron of TDP-43_M337V _mice, Nissl bodies became dispersed (Figure 7A) compared to NT mice (Figure 7B). There was disintegration of chromophil substance primarily within the cell bodies of the spinal cords of TDP-43_M337V _mice (Figure 7C) that was not observed in NT mice (Figure 7D). Ultrastructural analysis showed reduced cytoplasmic density and less cytoplasmic organelles in TDP-43_M337V _mice (Figure 7E) compared to NT mice (Figure 7F). These finding are indicative of chromatolysis and suggest that neurons in TDP-43_M337V _mice are undergoing perikaryal response to axonal degeneration.

### Hyperphosphorylated tau accumulation in TDP-43_M337V _and TDP-43_WT _mice

**Figure 8:**
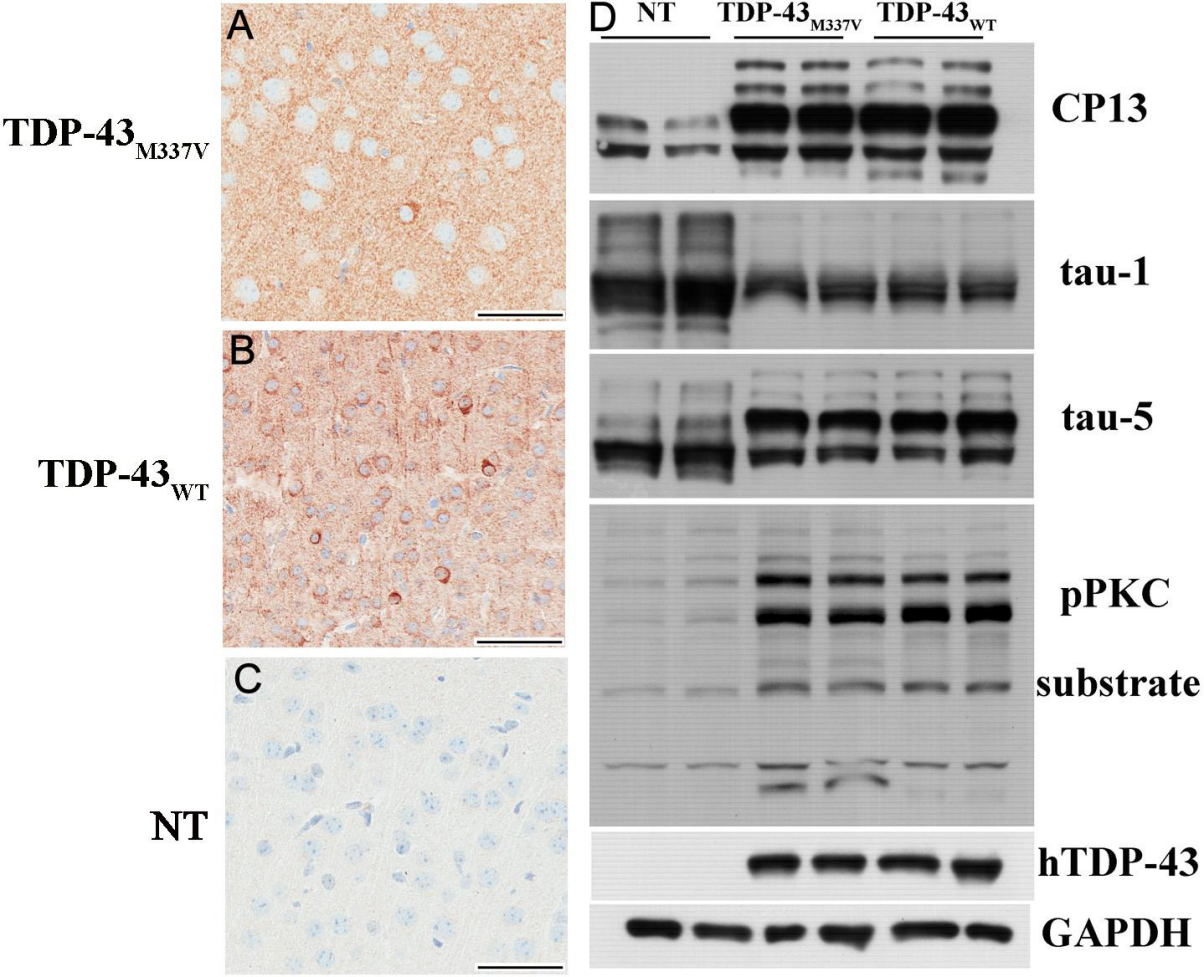
**Tau pathology in TDP-43_M337V _mice**. Immunohistochemistry of the cortices of (A) TDP-43_M337V _and (B) TDP-43_WT _mice showed elevated levels of phosphorylated tau (CP13) in both TDP-43_M337V _and TDP-43_WT _mice compared to (C) NT mice. (D) Western blotting of brain lysates from NT, TDP-43 _M337V _and TDP-43_WT _mice probed with CP13 antibody showed increases in murine tau phosphorylation at serine residues 202 in both lines of TDP-43 transgenic mice. Staining with tau-1 antibody showed reduced dephosphorylated tau levels in both lines of TDP-43 transgenic mice when compared to NT mice. Staining with antibody tau-5 showed that overall tau levels were equivalent across non-transgenic and transgenic mice. There was a dramatic increase of phospho-(Ser) PKC substrate in both TDP-43 transgenic lines indicating PKC activation that was not present in NT mice. Scale bars: 50 μM in A, B and C.

Abnormal, phosphorylated tau was found in the brains of both TDP-43_WT _and TDP-43_M337V _mice. Immunohistochemical analyses of the cortices of TDP-43_M337V _(Figure 8A) and TDP-43_WT _mice (Figure 8B) showed significantly elevated phosphorylated tau (CP13) immunoreactivity throughout the neuropil of the brain compared with NT mice (Figure 8C). Additionally, cytoplasmic tau accumulations were found in the TDP-43_WT _mice and much less frequently in the TDP-43_M337V _mice (Figure 8A, B). Western blots of brain lysates showed that the levels of phosphorylated tau (CP13) were significantly increased in both the TDP-43_WT _and TDP-43_M337V _mice (Figure 8D), while the level of dephosphorylated tau (Tau-1) was dramatically decreased. There was no significant change in the level of total tau (Tau-5) (Figure 8D). To explore the mechanism for tau phosphorylation, we examined protein kinases and phosphatases and found significant increases in phospho-(Ser) PKC substrate in both TDP-43_WT _and TDP-43_M337V _mice, indicating PKC activation may be responsible for such abnormal tau phosphorylation (Figure 8D). The levels of other tau kinases, such as GSK-3β and CDK5, and the tau phosphatase PP2A were not significantly changed in the TDP-43_M337V _and TDP-43_WT _mice (data not shown). Abnormally phosphorylated tau was rarely found in the spinal cord (data not shown). The regional distribution of hyperphosphorylated tau is summarized in Table 1.

## Discussion

In the current study, we generated and characterized a novel transgenic mouse model that overexpresses hTDP-43 carrying the M337V mutation under the mouse prion promoter. Homozygous TDP-43 M337V mice (referred to as TDP-43_M337V _mice) develop phenotypic and pathologic features including gait disturbances, gliosis, increased ubiquitination, TDP-43 truncation, phosphorylation, and both nuclear and cytoplasmic phosphorylated inclusions.

Importantly, the novel findings described in the current manuscript and the counterpart TDP-43_WT _mice manuscript [11], demonstrate expression of human TDP-43 at similar levels results in remarkably similar phenotypes and pathologies, suggesting that the phenotypes of both models are due to TDP-43 overexpression, and not specifically due to this mutation. Previously published findings regarding TDP-43 overexpression in mouse models strongly suggested that overexpression of TDP-43 itself is toxic; however, none of these studies have included mutant TDP-43 lines that were comparable in expression level and pattern, promoter system, and strain to WT TDP-43 counterparts [10-16]. In constitutive transgenic rat studies, Zhou and colleagues reported that mutant M337V TDP-43 appeared to be more toxic than wild-type TDP-43 [18] even though the animals expressed the hTDP-43 protein at similar levels. It is unclear why there is a discrepancy between our mutant and wild-type TDP-43 transgenic models and those in the rat. One possibility could be the difference of promoters utilized in the studies. Additionally, Zhou and colleagues did not look at endogenous rat TDP-43 levels in response to the hTDP-43 overexpression [18]; therefore, it is possible that differences in endogenous TDP-43 played a role in the rat phenotype. Moreover, in contrast to our hTDP-43 cDNA construct, the constitutive hTDP-43 construct utilized by Zhou and colleagues is a hTDP-43 minigene [18], which appears to contain 3'UTR and binding regions that have been shown recently to be required for autoregulation of TDP-43 [19,20]. Given this, it is possible that the hTDP-43 in the constitutive rat model is also autoregulated and that the presence of the mutation in the hTDP-43 minigene prevents autoregulation of the mutant, but not the wild-type, hTDP-43. Finally, in the TDP-43 rat models, there appears to be extensive hTDP-43 immunoreactivity in both the nucleus and the cytoplasm, regardless of mutation; whereas, TDP-43 mainly localized in the nuclei in both of our TDP-43_M337V _and TDP-43_WT _mice. The toxicity that we observe in our TDP-43 transgenic mice is therefore likely to be due, at least in part, to the impact of high TDP-43 levels on nuclear functions. Overexpression of TDP-43 in the nuclei of TDP-43 mice may interfere with interaction with its DNA and RNA targets, disrupt normal substrate metabolism and lead to neuronal dysfunction. Recent efforts to identify neuronal RNA targets of TDP-43 implicated in neurodegeneration led to the identification of FUS/TLS, progranulin, tau and ataxin1 and -2 and TDP-43 itself [20-22]. Additional studies are needed to explore the precise roles of TDP-43 DNA/RNA targets at work in these mouse models. Both TDP-43 LMW species and cytoplasmic TDP-43 are present in our TDP-43 mice, and they may also contribute to the pathologies and abnormal behavior.

Our results showed that overexpression of hTDP-43_M337V _can down regulate endogenous mouse TDP-43 levels, which is consistent with the property of TDP-43 autoregulation. TDP-43 can autoregulate its mRNA level, in part by directly binding to the 3'UTR of its own transcript, thereby triggering exosome-mediated degradation or nonsense-mediated RNA degradation [19,20]. The C-terminal region (a.a 321-366) of TDP-43 is required for autoregulation [19]. Recent reports allow for the possibility that mutation of TDP-43 within the C-terminus may affect the efficiency of autoregulation; however, the presence of the M337V mutant within hTDP-43 did not impair its ability to regulate endogenous mTDP-43. The loss of nuclear mTDP-43 in response to hTDP-43_M337V _overexpression has been proposed to play a role in the pathogenesis observed in another TDP-43 transgenic mouse model [14]. Considering the substantial homology between the hTDP-43_M337V _and mTDP-43 proteins, it would seem likely that hTDP-43_M337V _might functionally compensate for the loss of mTDP-43, but we cannot exclude the possibility that loss of nuclear mTDP-43 may contribute to the phenotype of hTDP-43_M337V _transgenic mice.

Abnormal mitochondrial accumulation has now been observed in both our TDP-43_M337V _and TDP-43_WT _mice [11] and in another wild-type TDP-43 overexpressing mouse model driven by Thy1.2 promoter [13], suggesting that TDP-43 can regulate mitochondrial dynamics. In addition to clustering of mitochondria, the size and the ultrastructural integrity of neuronal mitochondria was reduced in our TDP-43 mice compared with mitochondria of NT mice. In the TDP-43_WT _mice, we had previously identified changes in levels and the phosphorylation state for proteins that critically regulate mitochondrial fission and fusion and suggested that these changes may contribute to the abnormal aggregation and morphology of mitochondria. Surprisingly, we did not find similar changes in mitochondrial fission and fusion proteins in the TDP-43_M337V _mice, suggesting that mitochondrial aggregation observed in both TDP-43 models may result from another pathogenic processes, such as axonal degeneration.

On occasion, mitochondrial clusters within the neurons of the TDP-43_M337V _mice were associated with microtubules. TDP-43 may alter the microtubule-based mitochondrial transportation system and lead to both microtubule and mitochondrial aggregation and dysfunction. While there are a number of ways through which this could occur, it was interesting to find that the microtubule associated protein tau was abnormally hyperphosphorylated in the brain of both our TDP-43_WT _and TDP-43_M337V _mice. Tau functions to stabilize microtubules and to facilitate axonal transport. Abnormal phosphorylation of tau can reduce its ability to bind microtubules [23]. A number of kinases can phosphorylate tau, and one of these kinases, PKC, was activated in both TDP-43_M337V _and TDP-43_WT _mice. Tau hyperphosphorylation might be partially due to PKC activation in the mice; however, the paradigm through which TDP-43 overexpression resulted in tau hyperphosphorylation and/or PKC activation is still unclear. One possibility is that exacerbated TDP-43 autoregulation disrupts the homeostasis of other key proteins and eventually leads to dysfunction. Alternatively, PKC activation and tau hyperphosphorylation could be secondary or unrelated to mitochondrial clustering. There is a large body of evidence suggesting that axonal transport is disrupted in ALS [24,25], which includes evidence that SOD1 mutations impair axonal transport [26,27]. Interestingly, depletion of kinesin heavy chain (kif5B) results in mitochondrial perinuclear clusters similar to those described in our TDP-43 models [28].

One limitation of our model is that although many of the features are consistent with those observed in ALS, there are several features of this model that are incongruous with the human disease. Phenotypically, our mice show retarded growth from early life; whereas, humans with ALS typically show progressive disease after initially normal development into adulthood. While our TDP-43_M337V _mice show early lethality, the age at death for the mice is significantly younger in comparison to the lifespan of humans with ALS due to mutations in TDP-43. We did not see loss of nuclear TDP-43 immunoreactivity similar to that observed in affected neurons in ALS, even in cells with cytoplasmic TDP-43 inclusions. It is possible that mTDP-43 underwent redistribution as recently reported in another TDP-43 mouse model [14]; however, we are unable to confirm this due to unavailability of mTDP-43-specific antibody. We also observed considerable chromatolysis in the TDP-43_M337V _mice; however, chromatolysis is infrequent in end stage human ALS, and often only detected in cases with rapid disease progression [29]. TDP-43_M337V _mice also exhibit other pathologies not frequently seen in humans, such as abnormal mitochondrial aggregation. Interestingly, increased phospho-tau has been reported in ALS patients, especially those with cognitive impairment [30,31], which suggests that further analysis of a potential relationship between TDP-43 and tau in mouse models and in human cases might be warranted. The motor, biochemical, and pathological features observed in TDP-43_M337V _mice (line 4) were also noted in a second independent line of TDP-43_M337V _mice (line 6), suggesting that these features were not simply due to transgene insertional effects.

We observed some regional differences in our TDP-43 transgenic models, despite the relatively uniform expression of the TDP-43 proteins throughout the gray matter of the brain and spinal cord. For example, nuclear pTDP-43 inclusions, abnormal mitochondrial aggregation and chromatolysis were mainly observed in spinal cord, while cytoplasm pTDP-43 inclusions and tau phosphorylation were primarily seen in the brain. This indicates that the mechanisms regulating TDP-43 and being regulated by TDP-43 are likely to be different in the brain and spinal cord and warrant further investigation.

## Conclusion

In summary, this novel TDP-43_M337V _mouse model indicates that overexpression of hTDP-43_M337V _alone is toxic *in vivo*. TDP-43_M337V _mice recapitulate certain pathologic features seen in neurodegenerative diseases, including TDP-43 fragmentation, phosphorylation, aggregation, increased ubiquitination and gliosis. While these features could also indicate general neuronal dysfunction, the mice also exhibit down regulation of mouse TDP-43, abnormal mitochondria aggregation and abnormal tau phosphorylation. Because overexpression of mutant hTDP-43 produces phenotypes similar to the wild-type TDP-43 model, the mechanisms causing pathogenesis in the mutant model remain unknown. However, these results should not be surprising, given the mutant TDP-43 and wild-type TDP-43 biochemically are similar in human disease. As such, the TDP-43_M337V _mice may serve as a valuable tool for future studies of yet-to-be-examined disease pathways and the precise roles TDP-43 RNA targets play in neurodegeneration.

## Methods

### Generation of TDP-43_M337V _Transgenic Mice

The human wild-type TDP-43 cDNA was generated as previously described [11] and was inserted into the *Xho*I site of the pcDNA 3.1. To generate the M337V mutation, site directed mutagenesis was performed using Quikchange kit (Strategene). The primers used for the mutation were: 5'-CAGTTGGGGTATGGTGGGCATGTTAGC-3' and 5'-GCTAACATGCCCACCATACCCCAACTG-3'. After confirming the mutation by sequencing, the M337V TDP-43 cDNA was inserted into the *Xho*I site of MoPrP vector [17]. Following sequencing, the construct was linearized with *Not*I, gel purified and digested with β-agarose. DNA was filtered, concentrated and diluted to 3 ng/μl in microinjection buffer. The transgene was microinjected into fertilized C57BL/6 (B6) mouse eggs and re-implanted into pseudopregnant females. Eight founders were mated with B6 mice to determine germline transmission and establish expression levels. TDP-43 founder lines (line 4 and 6) that have similar transgene levels to the TDP-43_WT _mice we previously described [11], were used for all subsequent experiments. Homozygous mice were produced through a crossbreeding of transgenic mice from the same line. At no time were lines 4 and 6 intercrossed. Procedures were performed in accordance with the Mayo Institutional Animal Care and Use Committee.

### Genotyping

Transgenic mice were identified by PCR using hTDP-43-specific primers: 5'- TGGAGAAGTTCTTATGGTGCAGGTC-3' and 5'-GGTATTAGCCTATGGGGGACAC-3' against control actin-specific primers (5'-CGGAACCGCTCATTGCC-3' and 5'-ACCCACACTGTGCCCATCTA-3'). Homozygous mice were identified by Quantitative real-time PCR (see Quantitative real-time PCR section).

### Quantitative real-time PCR

Levels of human and mouse TDP-43 transcripts were determined via TaqMan^® ^Gene Expression Assays (Applied Biosystems, Carlsbad, CA). Total RNA was isolated either from tail samples for identification of homozygous mice or from brain or spinal cord tissue to determine the levels of human and mouse TDP-43 transcripts. TRIzol (Invitrogen, Carlsbad, CA) and Pure Link™ RNA Mini Kit (Invitrogen, Carlsbad, CA) were used for RNA extraction. 3 μg RNA were used to synthesize cDNA using the High Capacity cDNA Reverse Transcription Kit (Applied Biosystems, Carlsbad, CA). The qPCR assay used the following: hTDP-43 Hs00606522_m1, mTDP-43 Mm00523866_m and 18S rRNA Hs99999901_s1. The PCR was run on the ABI 7900 and data analyzed using Software RQ Manager 1.2 (Applied Biosystems, Carlsbad, CA).

### Tissue preparation

Sagittal half brain and spinal column were immersion fixed in 10% formalin for immunohistochemistry, and the other half brain was frozen on dry ice for biochemistry. After 24 hours, the spinal cord was removed from the vertebral column and fixed overnight.

### Western Blotting

Tissues were homogenized at 10 ml/g (volume/weight) in lysis buffer (50 mM Tris-HCl, pH 7.4, 300 mM NaCl, 1% Triton X-100, 5 mM EDTA, 2% SDS, PMSF, and protease and phosphatase inhibitor). Following centrifugation, supernatant was assessed by BCA assay (Pierce, Rockford, IL). Following western blotting, membranes were incubated with mouse monoclonal TDP-43 antibody, which was generated using amino acids 1 to 261 of hTDP-43 as the immunogen and was found to recognize amino acids 205-222 of hTDP-43 by epitope mapping [32]; rabbit polyclonal TDP-43 antibody against amino acids 288-441; mouse CP13; mouse Tau 5; mouse tau-1; mouse monoclonal glyceraldehyde-3-phosphate dehydrogenase (GAPDH) antibody; Phospho-(Ser) PKC Substrate Antibody; mouseDLP1 antibody; rabbit phospho-DLP1(Ser616) antibody; rabbit Fis1 antibody; or mouse mitofusin 1 antibody. See Additional file 3 for a complete list of primary antibodies used. Following incubation with an appropriate secondary antibody, immunoreactivity was visualized by ECL and exposure to film.

### Immunohistochemistry (IHC) and histochemistry

Tissues were embedded in paraffin, sectioned (5 μm thick) and mounted on glass slides. Sections were deparaffinized in xylene and rehydrated in a graded series of alcohol, followed by dH_2_0. Antigen retrieval was performed in a dH_2_O steam bath for 30 min. Tissues were immunostained with monoclonal TDP-43 antibody or antibodies toward pS403/S404-phosphorylated TDP-43, ubiquitin, glial fibrillary acidic protein (GFAP), ionized calcium-binding adaptor molecule 1 (IBA-1), cytochrome oxidase subunit IV (COX-IV), or phospho-tau (CP13; pS202 tau) using the DAKO Autostainer (Dako Auto Machine Corporation) and the DAKO EnVision+ HRP system. DAKO Liquid DAB Substrate-Chromogen system was the chromogen. See Additional file 3 for a complete list of primary antibodies used. After immunostaining, sections were briefly counterstained with hematoxylin to stain cell nuclei and coverslipped. Paraffin-embedded sections were also stained with hematoxylin and eosin.

### Electron Microscopy

Spinal cords from 4%-paraformaldehyde-perfused mice were immersed in 2.5% glutaraldehyde-0.1 M cacodylate buffer, post-fixed in 1% OsO_4_, dehydrated in alcohol and propylene oxide, and finally infiltrated and embedded in Epon 812. Ultrathin sections mounted on copper grids were stained with uranyl acetate and lead citrate. Images were obtained with a Gatan CCD camera using a Philips 208S electron microscope.

### Gait Analysis Methods

Front paws were painted red, and hind feet were painted blue with nontoxic paint. The mice were placed in a plastic tunnel with a strip of white paper covering the floor. At the end of the tunnel was an enclosed black box to encourage mice to cross the paper and into the box. The test was repeated until mice left at least five pairs of adjacent footprints.

### Statistics

One-way ANOVA with Tukey's posthoc analysis were used to compare measures among 3 groups. Student's t-test analysis was used to compare measures between 2 groups. Kaplan-Meier methods were used for survival analysis. For data presentation, normalized values were averaged and presented as mean ± standard error of means (SEM). Values of *p *< 0.05 were considered statistically significant.

## List of abbreviations

ALS: amyotrophic lateral sclerosis; FTLD-U: frontotemporal lobar degeneration with ubiquitin-positive inclusions; TDP-43: TAR DNA binding protein-43; hTDP-43: human TDP-43; mTDP-43: mouse TDP-43. pTDP-43: phospho-TDP-43.

## Competing interests

YX, YZ, JL, and LP are inventors of this and related mouse models; however, no royalties have been generated from this invention.

## Authors' contributions

YX and YZ performed experiments, data analysis and co-wrote the manuscript. XC performed experiments, WL performed Electron Microscopy study. CS edited the manuscript. JL, DWD and LP conceived of the study, participated in its design and coordination and edited the manuscript. All authors read and approved the final manuscript.

## Supplementary Material

Additional file 1 **Figure A1: Increased ubiquitin levels in TDP-43_M337V _mice but hTDP-43 itself is not ubiquitinated**. Human TDP-43 was immunoprecipitated from brain homogenates derived from nontransgenic and homozygous TDP-43_m337v _mice. Briefly, brain homogenates containing 500 μg protein were incubated with 1.5 μg mouse monoclonal TDP-43 antibody overnight at 4°C with gentle shaking. Protein G agarose was added for 4 h at 4°C then pelleted by centrifugation. Protein thus captured was eluted using sample loading buffer and resolved by SDS/PAGE for Western blot analysis. Shown are immunoblots of the inputs and the immunoprecipitated proteins, probed using an antibody to ubiquitin or to total TDP-43. Note that a marked increase in ubiquitin levels is observed in the homogenates derived from homozygous mice prior to immunoprecipitation. Nonetheless, the immunoprecipitated human TDP-43 is not immunopositive for ubiquitin. Arrow = IgG Heavy Chain. Click here for file

Additional file 2 **Figure A2: No mitochondrial fission and fusion protein changes in TDP-43_M337V _mice**. Immunoblot analysis of Ser616-phosphorylated DLP1, DLP1, Fis1, and mitofusin 1 (MFN1) expression level in brain lysates of nontransgenic(NT), hemizygous(Hemi), and homozygous(Homo) TDP-43_M337V _mice of both line 4 and line 6. There are no significant protein changes among different mice groups. Click here for file

Additional file 3 **Additional Table: Primary Antibody List**. Full list of the primary antibodies used in this study. Click here for file
