# Supplementary figures and images for: Expression of mutant TDP-43 induces neuronal dysfunction in transgenic mice
Source: Mol Neurodegener. 2011 Oct 26;6:73. doi: 10.1186/1750-1326-6-73 (PMC3216869; doi:10.1186/1750-1326-6-73)

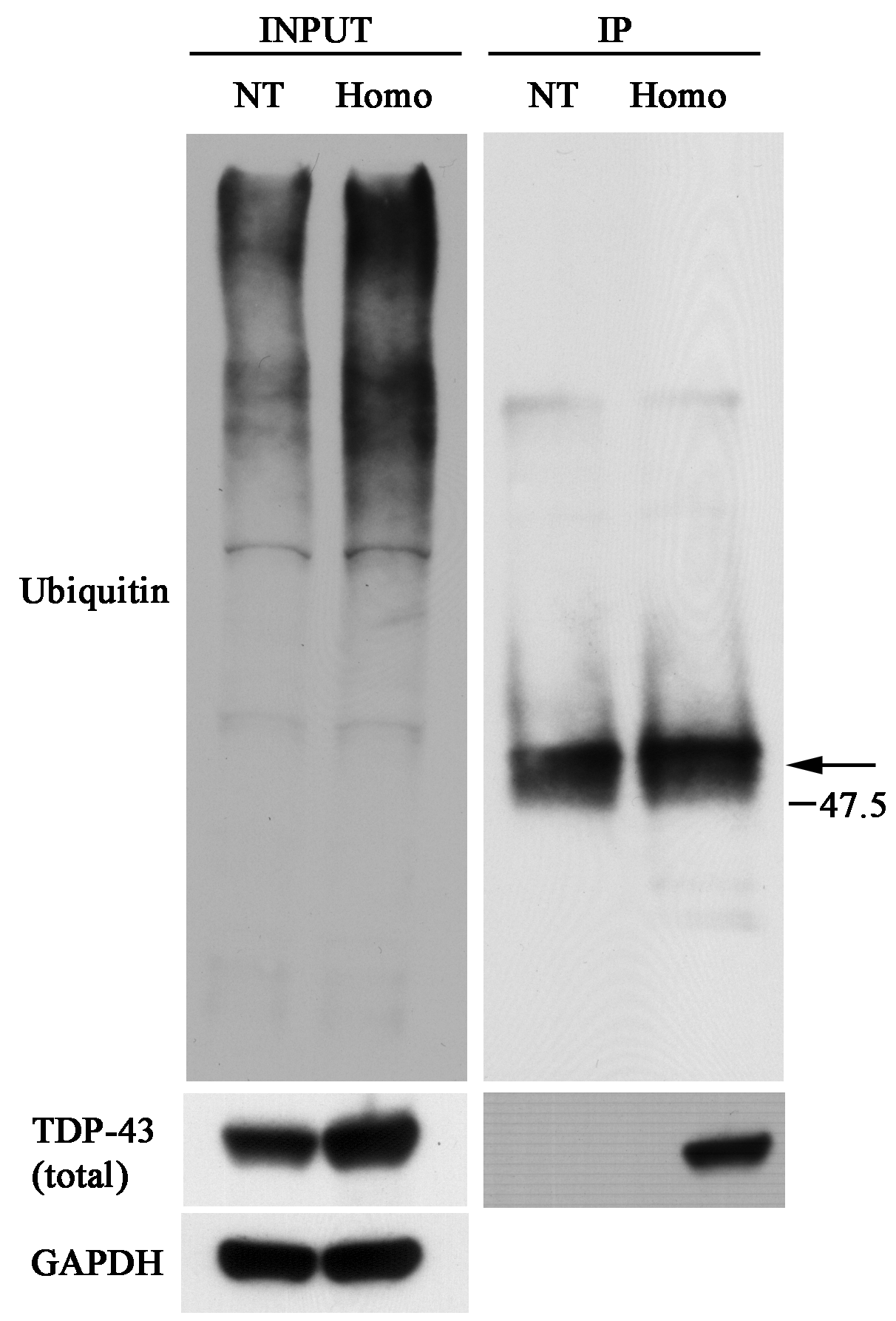

Supplement: Additional file 1 — Figure A1: Increased ubiquitin levels in TDP-43M337V mice but hTDP-43 itself is not ubiquitinated. Human TDP-43 was immunoprecipitated from brain homogenates derived from nontransgenic and homozygous TDP-43m337v mice. Briefly, brain homogenates containing 500 μg protein were incubated with 1.5 μg mouse monoclonal TDP-43 antibody overnight at 4°C with gentle shaking. Protein G agarose was added for 4 h at 4°C then pelleted by centrifugation. Protein thus captured was eluted using sample loading buffer and resolved by SDS/PAGE for Western blot analysis. Shown are immunoblots of the inputs and the immunoprecipitated proteins, probed using an antibody to ubiquitin or to total TDP-43. Note that a marked increase in ubiquitin levels is observed in the homogenates derived from homozygous mice prior to immunoprecipitation. Nonetheless, the immunoprecipitated human TDP-43 is not immunopositive for ubiquitin. Arrow = IgG Heavy Chain. [file 1750-1326-6-73-S1.TIFF]

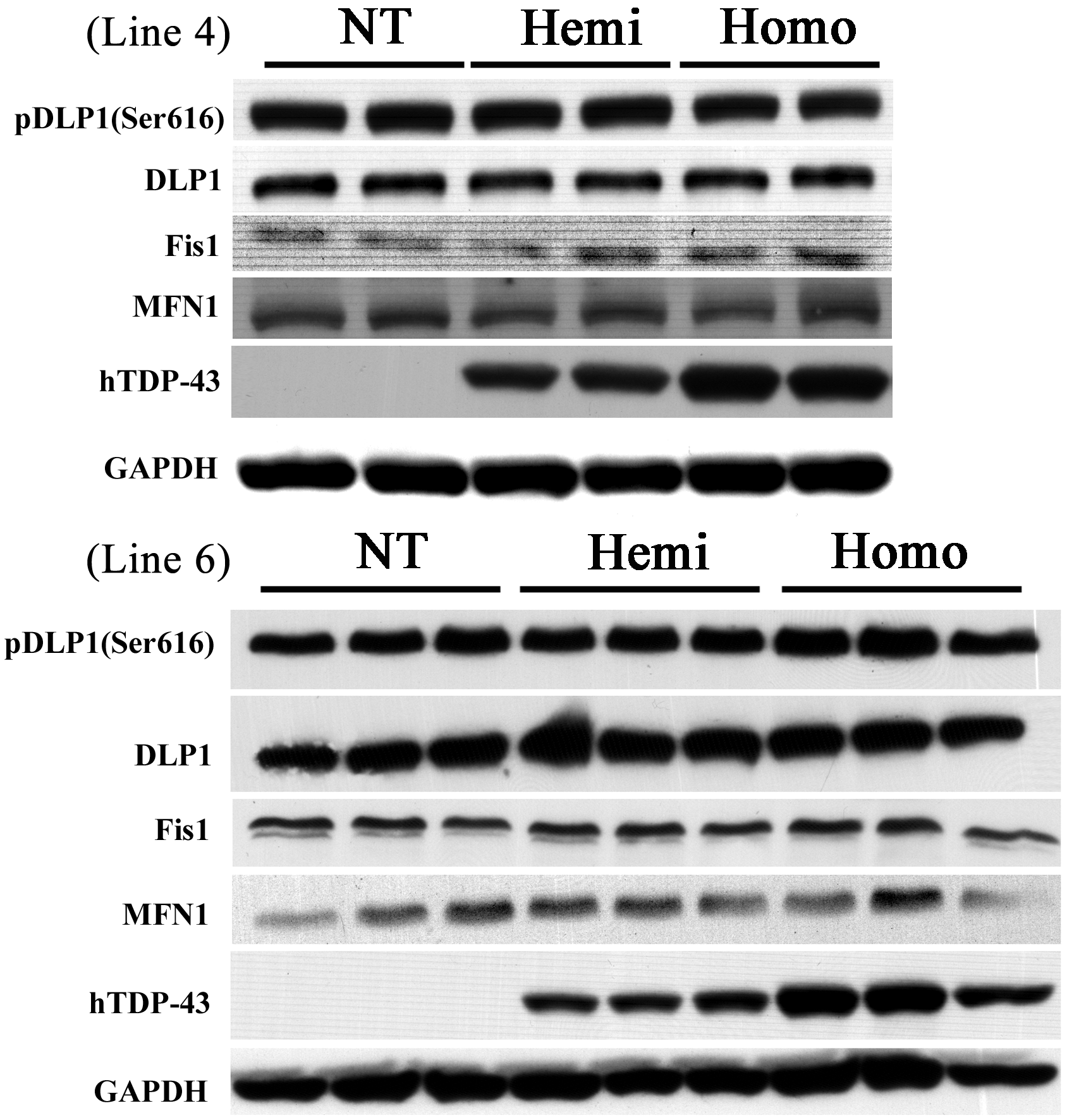

Supplement: Additional file 2 — Figure A2: No mitochondrial fission and fusion protein changes in TDP-43M337V mice. Immunoblot analysis of Ser616-phosphorylated DLP1, DLP1, Fis1, and mitofusin 1 (MFN1) expression level in brain lysates of nontransgenic(NT), hemizygous(Hemi), and homozygous(Homo) TDP-43M337V mice of both line 4 and line 6. There are no significant protein changes among different mice groups. [file 1750-1326-6-73-S2.TIFF]
