# Supplementary material for: Expression of mutant TDP-43 induces neuronal dysfunction in transgenic mice
Source: Mol Neurodegener. 2011 Oct 26;6:73. doi: 10.1186/1750-1326-6-73 (PMC3216869; doi:10.1186/1750-1326-6-73)
Supplement: Additional file 3 — Additional Table: Primary Antibody List. Full list of the primary antibodies used in this study. [file 1750-1326-6-73-S3.PDF]

Additional Table. Primary Antibody List

Antibody	Catologue #	Vendor	WB Dilution	IHC Dilution	Description
mouse monoclonal TDP-43 (hTDP-43)	2E2-D3	Novus Biologicals	1:1000	1:3000	recognizing human TDP-43 at amino acids 202-222
rabbit polyclonal TDP-43 (Total TDP-43)	12892-1-AP	ProteinTech Group	1:1000	1:3000	made to amino acids 260- 414 recognize both human and mouse TDP-43
rabbit phospho-TPD-43 (pS403/404)	TIP-PTD- P05	Cosmo Bio		1:2000	
ubiquitin	05-944	Chemicon/ Millipore		1:60000	
ubiquitin	Z0458	Dako	1:1000		
cytochrome c oxidase subunit IV (COX-IV)	ab16056	Abcam		1:3000	
GFAP	PU020-UP	Biogenex		1:2500	
Iba1	019-19741	Wako		1:2000	
GAPDH	A8634OH	Biodesign International	1:10000		
mouse CP13		Provided by Peter Davis Albert Einstein College of Medicine	1:1000	1:1000	recognizing pS202 tau
mouse Tau 5		Provided by Lester Binder Northwestern University	1:1000		recognizing total tau
mouse Tau1	MAB3420	CHEMICON	1:1000		anti-unphosphorylatedS202/205 tau
Phospho-(Ser) PKC Substrate Antibody	2261	Cell Signaling	1:500		
mouseDLP1 antibody	611112	BD	1:1000		
rabbit phospho-DLP1(Ser616)	3455	Cell Signaling	1:1000		
rabbit Fis1 antibody	IMG-5113A	IMGENEX	1:500		
mouse mitofusin 1 (MFN1)	H00055669-A01	Novus Biologicals	1:500		
